# A Low-Parameter Adaptive Framework Based on Gaussian Mixture Modeling for Detecting Weak Astrocytic Calcium Signals in Two-Photon Imaging

**DOI:** 10.3390/bioengineering13050528

**Published:** 2026-04-30

**Authors:** Jiameng Xu, Huiquan Wang, Shaofan Yang, Xiang Liao, Kuan Zhang, Guang Zhang

**Affiliations:** 1 School of Control Science and Engineering, Tiangong University, Tianjin 300161, China; xujiameng_96@foxmail.com (J.X.);; 2 Tianjin Key Laboratory of Quality Control and Evaluation Technology for Medical Devices, Tianjin 300161, China; 3 Brain Research Center and State Key Laboratory of Trauma and Chemical Poisoning, Third Military Medical University, Chongqing 400038, China; 4 Center for Neurointelligence, School of Medicine, Chongqing University, Chongqing 400030, China; xiang.liao@cqu.edu.cn; 5 Systems Engineering Institute, Academy of Military Sciences, People’s Liberation Army, Tianjin 300161, China

**Keywords:** two-photon imaging, astrocytic calcium signaling, weak signal detection, Gaussian mixture model, adaptive thresholding, biomedical image processing

## Abstract

Two-photon microscopy enables in vivo imaging of astrocytic Ca^2+^ activity, yet detecting weak, transient, and background-coupled signals remains challenging due to low signal-to-noise ratios and heterogeneous noise. Here, we propose a low-parameter, adaptive framework for detecting weak astrocytic Ca^2+^ signals in two-photon imaging. After short-window frame accumulation, static background suppression, and Gaussian smoothing to stabilize intensity statistics, signal candidates are identified via segment-wise Gaussian mixture modeling, temporal persistence masking, and adaptive threshold updates. In simulated videos, the proposed method improved the Dice coefficient from 0.06 to 0.77 and increased the reference SNR from −9.82 to 3.40 dB. In in vivo recordings, the local SNR increased from 5.58 to 7.28 dB. Compared with fixed thresholding, AQuA, and AQuA2, our method was more robust under high-noise conditions while requiring only three user-defined parameters (minimum area, minimum duration, and an initialization coefficient). This framework provides an interpretable and computationally practical front-end module for the robust extraction of astrocytic Ca^2+^ signal in low-SNR two-photon imaging.

## 1. Introduction

Astrocytes are widely distributed throughout the brain and communicate with neurons and the surrounding microenvironment through intracellular Ca^2+^ signaling, making astrocytic calcium activity an important readout for investigating brain network function and neurological disorders [1,2,3,4]. Two-photon microscopy provides subcellular spatial resolution and high temporal resolution for in vivo imaging of astrocytic calcium transients [5,6,7]. However, reliable detection and segmentation remain challenging because astrocytic events are often weak, transient, and strongly background-coupled, while the acquired images typically exhibit low signal-to-noise ratios (SNRs) and heterogeneous noise [8].

Early strategies, such as temporal and spatial smoothing, often suppress noise at the expense of fine event details and fail to preserve transient event-propagation fidelity [9]. Although denoising algorithms—including wavelet transforms, non-local means filtering, matrix decomposition, and deep learning—have shown excellent performance in neuronal imaging, astrocytic signals are weaker and more strongly coupled with background and noise, complicating the balance between spatiotemporal fidelity and noise suppression [9,10,11]. Furthermore, the complex spatiotemporal dynamics of astrocytic activity limit the applicability of regions-of-interest (ROI)-based analyses.

Recent advancements have attempted to address these challenges but exhibit notable limitations in practical engineering applications. For instance, Barbera et al. [12] applied ΔF/F_0_ normalization and slow-drift baseline removal; however, this primarily addresses temporal-domain drift without accounting for interference from static spatial structures, potentially leading to false detections in scenarios with strong background–signal coupling. Matrix decomposition frameworks like Minian [13] achieve joint separation of signals and noise, yet their parameter selection relies heavily on visual inspection, and their transferability to fast two-photon imaging remains limited. Deep-learning-based approaches, such as DeepCAD [14] and Noise2Void [15], as representative self-supervised denoising methods for two-photon calcium imaging, have demonstrated powerful noise suppression performance as preprocessing modules in the analytical workflow. Related learning-based denoising strategies have also been explored in other noisy biomedical imaging modalities, such as ultrasound localization microscopy, where deep neural networks were used to enhance microbubble signal recovery under severe noise conditions [16]. However, the imaging physics, target structures, and downstream analytical objectives differ substantially from those of two-photon astrocytic Ca^2+^ event detection. Nevertheless, these models do not include built-in modules for recognizing and segmenting calcium activity and therefore must be combined with downstream detection algorithms to complete the analytical pipeline. Meanwhile, these methods require high computational resources, demand extensive training data for optimal performance, and may generate artificial structures during denoising that compromise the interpretability of subsequent signal analysis. From the viewpoint of astrocytic Ca^2+^ signal analysis, existing pipelines can be broadly grouped into ROI-based summarization, event-oriented analytical frameworks, and denoising-centered preprocessing modules. ROI-based analyses are less suitable for irregular and propagative astrocytic Ca^2+^ activity, whereas AQuA and AQuA2 are representative event-oriented frameworks designed for spatiotemporal event parsing. By contrast, DeepCAD and Noise2Void mainly function as denoising front ends rather than end-to-end event detectors. The present work is therefore positioned as a low-parameter front-end framework for weak-signal event extraction under low-SNR, strongly background-coupled two-photon imaging. Recent astrocyte-oriented analysis tools further indicate that current methods are diverging toward different analytical goals: astroCaST emphasizes event detection and clustering, STARDUST [17] provides an ROA-based workflow for regional calcium-dynamics analysis, and AQuA/AQuA2 focus more strongly on spatiotemporal event parsing; by contrast, the present work is positioned as a low-parameter front-end framework for weak-signal extraction under low-SNR, strongly background-coupled two-photon imaging conditions.

These limitations highlight a practical need for a detection framework that is robust to heterogeneous noise, tolerant of strong background coupling, and sufficiently parameter-efficient for routine use in two-photon astrocytic imaging. From a bioengineering perspective, such a framework should retain transient event structure while remaining interpretable, reproducible, and computationally practical for large-scale image analysis.

To address this problem, we developed a low-parameter adaptive detection framework for weak astrocytic calcium signals in two-photon imaging. In this framework, frame accumulation, static background subtraction, and Gaussian spatial smoothing are used as supporting preprocessing steps to stabilize input statistics and improve contrast for subsequent analysis. The core detection stage applies segment-wise Gaussian mixture model (GMM) estimation, temporal masking, and adaptive global/local threshold updating to identify temporally coherent signal regions under complex noise conditions. The framework requires only three user-defined parameters, namely the minimum area, minimum duration, and a global threshold initialization coefficient, thereby reducing manual tuning. The main methodological contribution of this work is a coupled low-parameter detection core for weak astrocytic calcium signals, rather than any single preprocessing or visualization step. Within this core, segment-wise GMM estimation serves as the primary signal-background separation mechanism, adaptive threshold updating acts as a dynamic sensitivity-control mechanism under time-varying noise and signal conditions, and temporal masking serves as a temporal-consistency constraint that suppresses isolated fluctuations. Accordingly, the key novelty of the proposed framework lies in the coordinated integration of local distribution modeling, adaptive decision boundary updating, and temporal persistence enforcement within a low-parameter detection pipeline. The results show improved segmentation fidelity in simulated data and improved image-quality metrics in in vivo data, supporting the framework as a practical, interpretable front-end analysis approach for high-noise astrocytic calcium imaging.

## 2. Materials and Methods

### 2.1. Image Preprocessing

The preprocessing pipeline was designed to stabilize input statistics and improve contrast for the subsequent adaptive detection stage, rather than to serve as the final signal extraction step. First, frame accumulation was applied to the raw video sequence to enhance weak calcium transients and suppress additive independent noise. Based on the reported minimum duration of astrocytic calcium events (approximately 333 ms [18]) and the acquisition rate of 40 Hz, the accumulation window length was set to 13 frames with a sliding interval of 1 frame [19]. This short-window accumulation was selected to match the expected event timescale, thereby reducing stochastic Poisson–Gaussian noise while limiting loss of temporal resolution and preserving transient structure. Because excessively large windows may blur short-lived events, the accumulation length should be selected according to the acquisition rate and the expected duration of calcium events.

Before accumulation, the original 8-bit grayscale sequence was converted to 16-bit format to prevent overflow and saturation during summation. Since each raw pixel ranges from 0 to 255, the theoretical maximum accumulated intensity for an m-frame window is 255× m; thus, any accumulation over more than one frame exceeds the 8-bit upper limit. Without this conversion, accumulated intensities would be clipped, distorting weak signal–background contrast and biasing subsequent intensity-based operations. The 16-bit representation, therefore, preserves the dynamic range produced by frame accumulation and improves the numerical stability of the subsequent preprocessing and adaptive detection stages.

After accumulation, a static background image was estimated by temporal averaging, and each frame was subjected to background subtraction and normalization to retain the dynamic signal and residual noise components [20]. The resulting 16-bit sequence was then processed using an isotropic Gaussian filter (σ = 1.0) and mirror-padded at the image boundaries to further suppress high-frequency noise and reduce baseline fluctuations [21]. Implementation details of the preprocessing procedures are provided in the Supplementary Materials.

### 2.2. Adaptive Dynamic Detection Framework Based on Gaussian Mixture Modeling

**Figure 1:**
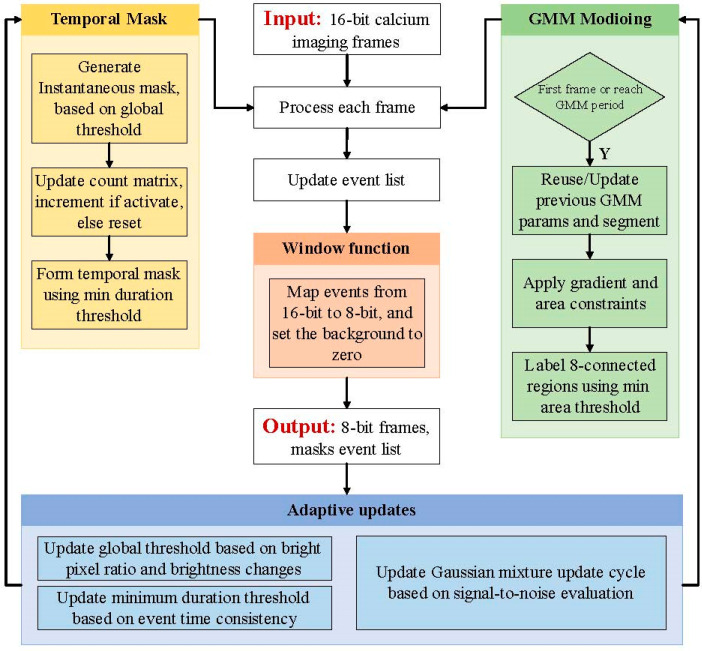
Workflow of the adaptive dynamic detection algorithm based on adaptive thresholding and Gaussian mixture modeling.

After the supporting preprocessing steps described in Section 2.1, the proposed framework takes the preprocessed 16-bit calcium imaging sequence as input and generates an 8-bit output sequence for visualization and downstream analysis. All data processing and analysis were performed using Python (version 3.11, Python Software Foundation, Wil-mington, DE, USA). The methodological novelty lies in the core detection stage rather than in the final intensity remapping step. Among the three detection-related components, segment-wise GMM estimation is the primary element enabling weak-signal detection, because it performs data-adaptive separation of signal-related intensity peaks from heterogeneous background distributions. Adaptive threshold updating is the second key component, dynamically adjusting the decision boundary based on frame-level statistics and temporal similarity under time-varying noise conditions. Temporal mask construction mainly serves as a temporal-consistency constraint, suppressing isolated fluctuations and reducing false positives. The overall workflow, therefore, comprises four coupled modules: temporal mask construction, segment-wise GMM estimation, adaptive threshold updating, and window-function transformation, with the last module used only for output mapping and display compatibility. Only three user-defined parameters are required for stable operation, namely the minimum calcium signal area, the minimum duration, and the global threshold initialization coefficient. This low-parameter design reduces manual tuning and improves robustness across datasets acquired under different experimental conditions. The detailed mathematical formulations of the four modules are presented in Section 2.2.1, Section 2.2.2, Section 2.2.3 and Section 2.2.4, and the overall workflow is illustrated in Figure 1.

#### 2.2.1. Temporal Mask Construction

For the *t*-th frame $I_{t}\left(x,y\right)$, the frame-wise mean $\mu_{t}$ and standard deviation $\sigma_{t}$ are calculated, as shown in Equation (1). These statistics provide a global description of the current intensity distribution and are used to determine whether individual pixels exhibit abnormally high intensity relative to the frame background.

(1) $$\mu_{t}=\frac{1}{XY}\sum_{h,w}I_{t}\left(x,y\right),\;\sigma_{t}=\sqrt{\frac{1}{XY}\sum_{x,y}{\left(I_{t}\left(x,y\right)-\mu_{t}\right)}^{2}}$$

where X and Y are the image width and height, respectively. Using a real-time-updated global threshold $\theta_{g}^{\left(t\right)}$, an instantaneous mask $M_{t}^{inst}$ is generated according to Equation (2). Pixels exceeding the threshold are marked as candidate active pixels in the current frame. This step provides an initial screening of bright regions but does not yet distinguish temporally coherent calcium activity from isolated fluctuations.

(2) $$M_{t}^{inst}\left(x,y\right)=\left\{\begin{array}{l} 1,I_{t}\left(x,y\right)>\mu_{t}+\theta_{g}^{\left(t\right)}\sigma_{t}\\ 0,otherwise \end{array}\right.$$

To incorporate temporal persistence, a count matrix is updated according to Equation (3). This matrix records the number of consecutive frames in which each pixel remains active. In this way, transient isolated noise can be suppressed, while pixels showing sustained activation over multiple frames are retained.

(3) $$C_{t}\left(x,y\right)=\left\{\begin{array}{l} C_{t-1}\left(x,y\right)+1,M_{t}^{inst}\left(x,y\right)=1\\ 0,otherwise \end{array}\right.$$

The temporal mask $M_{t}^{TCM}$ is then defined according to Equation (4). Here, the local duration threshold determines the minimum number of consecutive active frames required for a pixel to be preserved in the temporal mask, i.e., a pixel is retained only when its consecutive-activation count reaches or exceeds the local duration threshold. In this study, the initial minimum duration criterion was set to 5 frames, corresponding to 0.125 s at 40 Hz. The same initial value was used for both the simulated and in vivo datasets in this work, because both were analyzed under the same temporal sampling condition. Thus, the parameter was not re-tuned separately for each dataset in the present study. More generally, this parameter should be interpreted as a minimum temporal persistence requirement and scaled to the acquisition rate and the expected minimum duration of calcium events when the framework is applied to datasets with different temporal resolutions or signal kinetics. The resulting binary temporal mask defines the pixel participation range for subsequent segment-wise estimation and adaptive threshold updating.

(4) $$M_{t}^{TCM}\left(x,y\right)=\left\{\begin{array}{l} 1,C_{t}\left(x,y\right)\geq \theta_{l}^{\left(t\right)}\\ 0,otherwise \end{array}\right.$$

#### 2.2.2. Segment-Wise Estimation via Gaussian Mixture Model

A Gaussian mixture model (GMM) is applied to the gray-level distribution of each frame for unsupervised decomposition, mapping statistically bright peaks to candidate active calcium signal regions. Segment-wise estimation is initialized at the first frame and subsequently updated at intervals defined by the estimation period.

The t-th frame $I_{t}\left(x,y\right)$ is flattened into a one-dimensional vector $h\in R^{XY}$, assuming its probability density $p\left(h\right)$ follows candidate Gaussian mixture models with *K* = 1, 2, or 3 components, corresponding to background-dominant, intermediate/transition intensities, and signal-enriched bright components, as shown in Equation (5).

In this formulation, each component is characterized by its weight, mean, and standard deviation. The model parameters are estimated using the Expectation–Maximization algorithm. If all pixel intensities are identical, the distribution degenerates to a single-component case.

(5) $$p\left(h\right)=\sum_{k=1}^{K}\pi_{k}N\left(h|\mu_{k},\sigma_{k}^{2}\right),1\leq K\leq 3,\sum_{k=1}^{K}\pi_{k}=1$$

where $\pi_{k}$ is the weight, and $\mu_{k},\sigma_{k}$ are the mean and standard deviation of the *k*-th component. The parameters $\left\{\pi_{k},\mu_{k},\sigma_{k}\right\}$ are estimated using the Expectation–Maximization algorithm.

After sorting components in ascending order of $\mu_{k}$, the rightmost component is defined as the active calcium signal peak, while the others are treated as background. For each component k, segment processing is performed according to Equation (6). This step defines the gray-level interval and the corresponding intensity threshold for each component, thereby allowing local intensity structure to be analyzed in a data-adaptive manner.

(6) $$S_{k}=\left(L_{k},H_{k},\tau_{g}^{\left(k\right)},\tau_{a}^{\left(k\right)}\right)$$

where $\left[L_{k},H_{k}\right]$ denotes the gray-level interval, truncated from $\mu_{k}\pm 2\sigma_{k}$ to the actual gray-level range. $\tau_{g}^{\left(k\right)}$ is the intensity threshold, defined as $\tau_{g}^{\left(k\right)}=\max\left(2,\beta_{k}\sigma_{k}\right)$. For signal or sub-signal components, $\beta_{k}=0.5$ for background components, $\beta_{k<K}=2$. $\tau_{a}^{\left(k\right)}$ is the area threshold, applied to substructures within signal regions, with the minimum area in this study set to 29 pixels (1 pixel = 0.187 μm). Background noise components are treated separately to suppress the influence of dominant non-signal structures.

(7) $$\tau_{a}^{\left(k\right)}=\left\{\begin{array}{l} \min\text{\_}area,k=K\\ 10\cdot \min\text{\_}area,k<K \end{array}\right.$$

A binary mask $M_{K}$ for the active calcium signal is then generated within the signal peak range $\left[L_{k},H_{k}\right]$:

(8) $$M_{K}\left(x,y\right)=\left\{\begin{array}{l} 1,L_{k}\leq I_{t}\leq H_{k}\\ 0,otherwise \end{array}\right.$$

An 8-connected component labeling is applied to $M_{K}$, retaining only regions $c_{i}$, meeting both area $\geq \tau_{g}^{\left(k\right)}$ and gradient criteria $\geq \tau_{a}^{\left(k\right)}$.

The union of $M_{event}\left(x,y\right)$ with all $c_{i}$ (for i ranging over the width) and the corresponding original intensity responses $I_{t}\left(M_{event}\right)$ are established.

For reproducibility, the implementation details of the segment-wise GMM are specified as follows. At each estimation step, the preprocessed frame is flattened into a one-dimensional gray-level vector and fitted using candidate Gaussian mixture models with *K* = 1, 2, or 3 components. The optimal model order is selected using the Bayesian information criterion. The expectation–maximization algorithm is initialized by k-means-based initialization and terminated when the log-likelihood increment is smaller than 10^−3^, or when the maximum number of iterations reaches 100. After convergence, the fitted components are sorted by their means in ascending order, and the component with the largest mean is defined as the active calcium signal peak, whereas the remaining components are treated as background-related components. In degenerate cases with nearly constant intensity values, the distribution is treated as a single-component case. These settings were fixed throughout all experiments and were not treated as user-defined parameters.

#### 2.2.3. Adaptive Threshold Updating

The adaptive threshold updating step is heuristic rather than posterior-probability-based or likelihood-ratio-based. Specifically, it is a statistics-driven empirical update strategy designed to regulate the decision boundary under time-varying signal prevalence and heterogeneous noise conditions. Because the preprocessed sequence exhibits frame-dependent intensity distributions and temporal nonstationarity, it is difficult to specify a robust, closed-form probabilistic decision rule for all frames. The present design, therefore, uses frame-level bright-pixel prevalence, temporal fluctuation, inter-frame mask consistency, and frame-specific SNR as empirical control variables for dynamic threshold regulation.

It uses frame-level bright-pixel prevalence, temporal fluctuations, inter-frame Jaccard similarity, and frame-specific SNR as empirical control variables to regulate the global threshold, local duration threshold, and GMM update period under heterogeneous and time-varying imaging conditions.

The global threshold $\theta_{g}^{\left(t\right)}$ for the *t*-th frame is first updated according to Equation (9) using the proportion of bright pixels in the current frame. This quantity serves as a frame-level empirical estimate of current signal prevalence.

(9) $$\rho_{t}=\frac{1}{XY}M_{t}^{TCM}\left(x,y\right)$$

The fluctuation term is then calculated, and the global threshold is further updated using Equation (10). This empirical correction allows the threshold to respond not only to the absolute proportion of bright pixels but also to its temporal variation.

(10) $$\theta_{g}^{\left(t\right)}=\theta_{g}^{\left(t-1\right)}\times \left\{\begin{array}{l} 1.2,\Delta \rho_{t}<0.001\\ 0.8,\Delta \rho_{t}\geq 0.001 \end{array}\right.$$

where $\Delta \rho_{t}=\left|\rho_{t}-\rho_{t-1}\right|$. The local threshold $\theta_{l}^{\left(t\right)}$ is updated using the inter-frame Jaccard index, as defined in Equation (11). The Jaccard index quantifies the overlap between adjacent masks and therefore measures the temporal consistency of the detected signal pattern.

(11) $$J_{t}=\frac{\left|M_{t}^{TCM}\cap M_{t-1}^{TCM}\right|}{\left|M_{t}^{TCM}\cup M_{t-1}^{TCM}\right|+\varepsilon },\;\varepsilon \;=\;10^{-9}$$

where $\varepsilon \;=\;10^{-9}$ is added to the denominator for numerical stability, to avoid division by zero when the union of two adjacent masks is empty. Because the denominator represents the pixel count of the union set and is therefore at least 1 in non-degenerate cases, this term is negligibly small and does not materially alter the Jaccard value under normal conditions.

Based on this similarity measure, the local duration threshold is updated according to Equation (12). In this way, temporally persistent candidates are preserved more readily, whereas unstable or weakly repeated fluctuations are suppressed.

(12) $$\theta_{l}^{\left(t\right)}=\left\{\begin{array}{l} \min\left(1.3\cdot \theta_{l}^{\left(t-1\right)},0.5\right),J_{t}>0.7\\ \max\left(0.9\cdot \theta_{l}^{\left(t-1\right)},0.2\right),otherwise \end{array}\right.$$

The estimation period used for segment-wise GMM updating is further adjusted through a smoothing coefficient. First, the signal-to-noise ratio is calculated according to Equation (13), where the numerator is the mean gray value of pixels inside the mask and the denominator is the standard deviation of pixels outside the mask. This ratio provides a frame-specific measure of signal prominence relative to the surrounding background.

(13) $$SNR_{t}=\frac{mean\left(I_{t}\left[M_{t}^{TCM}\right]\right)}{std\left(I_{t}\left[\text{\textasciitilde{}}M_{t}^{TCM}\right]\right)}$$

The numerator is the mean grayscale of the pixels inside the mask, and the denominator is the standard deviation of the pixels outside the mask.

The smoothing coefficient is then calculated as $\alpha^{\left(t\right)}=clip_{\left[0.85,0.99\right]}\left(1-\frac{1}{SNR_{t}}\right)$, and the estimation period N is updated according to Equation (14). This empirical adaptive update allows the segment-wise estimation interval to vary with signal quality, thereby improving the stability of GMM fitting under different noise levels and scene conditions.

(14) $$N=\max\left(1,round\left(\frac{1}{1-\alpha^{\left(t\right)}}\right)\right)$$

#### 2.2.4. Window-Function Transformation

After adaptive detection, the identified active calcium signal intensities are mapped from 16-bit to 8-bit values to facilitate display and storage while preserving relative structural detail. Direct display of 16-bit data may compress visually relevant contrast, whereas the present window-function transformation provides a controlled dynamic-range compression for detected signal regions.

As shown in Equation (15), background pixels are set to zero. $O_{t}^{8bit}\left(x,y\right)=0,\left(x,y\right)\notin M_{event}$, and the detected active signal intensities are linearly compressed into the 8-bit range. Here, the frame accumulation window length is incorporated to compensate for the amplitude scaling introduced during preprocessing.

(15) $$O_{t}^{8bit}\left(x,y\right)=round\left(\frac{I_{t}\left(x,y\right)}{\min(m\cdot 255,2^{16}-1)}\cdot 255\right),\left(x,y\right)\in M_{event}$$

where m is the frame accumulation window length.

This window-function transformation is monotonic within the detected signal mask, meaning that the relative ordering of intensities and the local gradients are preserved after mapping. Therefore, the transformation does not alter event boundaries or spatial structure, but only compresses the dynamic range for 8-bit representation. By compensating for the accumulation-induced intensity scaling, the transformation also helps prevent saturation and maintain consistent contrast across frames.

### 2.3. Evaluation Metrics

To evaluate the framework, we employed three metrics: noise estimation, signal-to-noise ratio (SNR), and segmentation overlap. Due to the availability of ground truth only in simulated data, evaluation was conducted at two levels: (1) for simulated data, assessing both detection accuracy and image quality; (2) for in vivo data, focusing on noise suppression and signal enhancement as indirect proxies for performance.

#### 2.3.1. Noise Estimation

Block Statistics Method [22]: The image is divided into fixed-size, non-overlapping blocks. The variance of the lowest 10% of blocks is computed, and the median of those variances is taken as the noise estimate for that frame. The average over the entire sequence is reported.

Wavelet Transform Method [23]: Each frame undergoes a one-level discrete wavelet decomposition. The median absolute deviation (MAD) of the highest-frequency diagonal sub-band coefficients is used to estimate the noise standard deviation, which is then averaged over the sequence.

#### 2.3.2. Signal-to-Noise Ratio (SNR)

Local-Statistics-Based SNR [24]: Computed from local mean filtering and residuals, suitable for real imaging data without ground truth.

Reference SNR [25]: For simulated data, calculated from the mean squared error (MSE) between the ground-truth and estimated frames, relative to the signal power.

#### 2.3.3. Dice Coefficient [26]

Measures the spatiotemporal overlap between the detected results and the ground-truth mask, ranging from 0 to 1, with higher values indicating more accurate segmentation.

Implementation details are provided in the Supplementary Materials.

### 2.4. Experiments

The proposed framework was evaluated using both simulated datasets and in vivo two-photon calcium imaging data from the mouse primary somatosensory cortex (S1). The two types of datasets served different validation purposes. The simulated datasets were used for standardized evaluation with event-level ground truth, enabling direct assessment of segmentation fidelity and signal recovery. The in vivo datasets were used to assess robustness under real biological imaging conditions, where strong background coupling, heterogeneous noise, and the absence of event-level ground truth make detection substantially more challenging.

**Figure 2:**
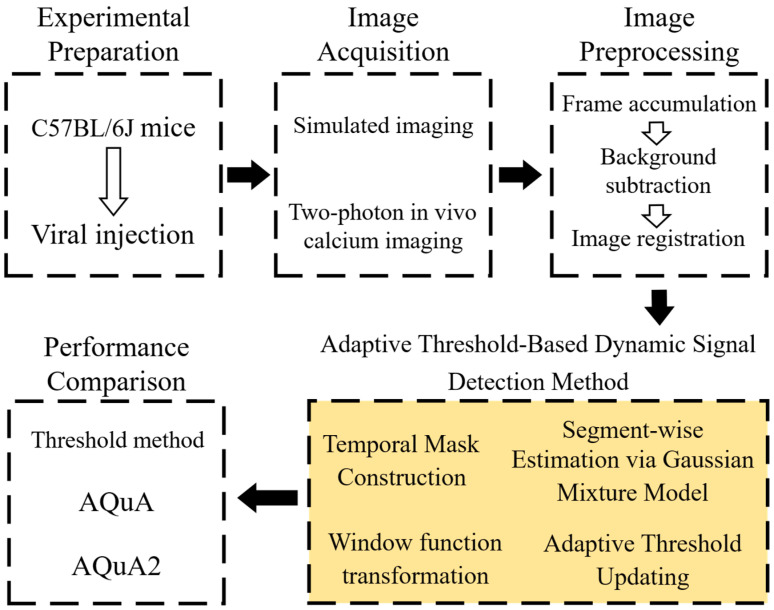
Experimental workflow for validating the proposed adaptive dynamic detection method. Solid arrows indicate the main processing flow, hollow arrows denote sequential operations within each module.

In total, the validation experiments included seven simulated two-photon videos and in vivo recordings from seven mice. The complete experimental workflow comprised image acquisition or dataset construction, preprocessing, adaptive GMM-based dynamic detection, and quantitative evaluation. The detailed workflow is shown in Figure 2, where the yellow-shaded box highlights the processing stage corresponding to the proposed framework.

#### 2.4.1. Simulated Imaging

To enable standardized and reproducible evaluation, simulated two-photon datasets were constructed to mimic the main components of astrocytic calcium imaging, including a static cellular skeleton background, dynamic calcium signals, and multi-source imaging noise. The dominant noise sources in two-photon imaging include shot/dark noise, which can be approximated as Poisson-distributed, and read noise, which is commonly modeled as Gaussian-distributed; accordingly, the overall noise characteristics were approximated using a Poisson–Gaussian model [27,28,29]. Simulated datasets were generated by combining programmatically defined motion signals with real image-derived structural background information [30].

**Figure 3:**
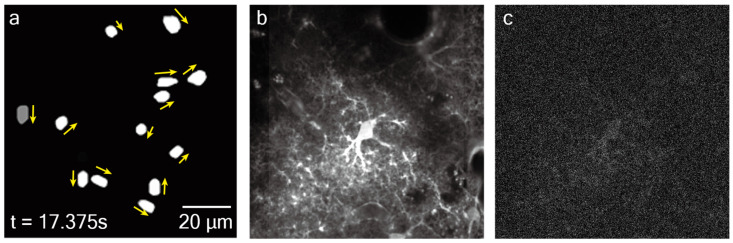
Construction of simulated two-photon calcium imaging datasets. (**a**) Moving light spots used to simulate active calcium signals (t = 17.375 s), with yellow arrows indicating the motion direction of the calcium signal in the current frame. (**b**) Real static cellular skeleton background extracted from actual imaging data. (**c**) Simulated two-photon microscopy imaging of astrocytic calcium, obtained by fusing (**a**,**b**) and adding Poisson and Gaussian noise.

First, a custom program was used to generate moving light spots over time to simulate the spatiotemporal evolution of calcium events (duration: 1 min; total events: 97; Figure 3a). Yellow arrows indicate the motion direction of representative simulated signals. Second, the dynamic signal was superimposed onto a static cellular skeleton background extracted by temporal averaging of a 10-minute real two-photon imaging sequence acquired at 40 Hz (Figure 3b). During image fusion, the motion-signal weight was set to 0.2 and the background weight to 0.8 to ensure that the structural occlusion effect of the cellular skeleton was preserved while weak signals remained detectable. Finally, mixed Poisson–Gaussian noise was added to generate the final simulated two-photon images (Figure 3c). Using this procedure, seven simulated two-photon videos were constructed for quantitative evaluation. Detailed fusion procedures and parameter settings are provided in the Supplementary Materials.

These simulated datasets were designed as a controlled but biologically informed approximation of astrocytic two-photon imaging, intended to preserve key features relevant to weak-signal detection—namely, dynamic event motion, strong structural background coupling, and mixed Poisson–Gaussian noise—rather than to reproduce the full biological complexity of in vivo astrocytic Ca^2+^ activity.

#### 2.4.2. In Vivo Two-Photon Ca^2+^ Imaging in Mice

**Figure 4:**
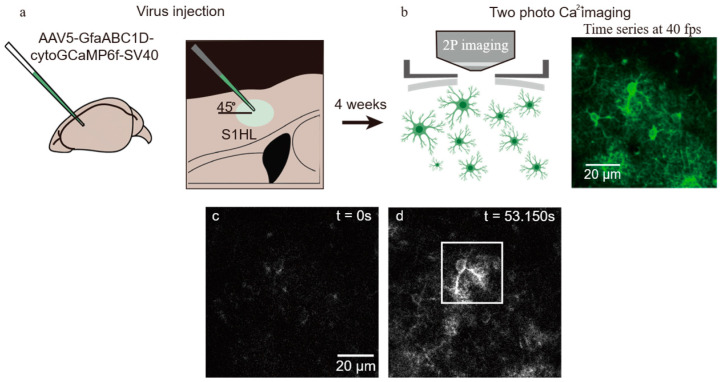
In vivo two-photon calcium imaging experiment in the mouse cortex. (**a**) Illustration of the viral injection procedure. (**b**) Two-photon imaging process, with green pseudocolor highlighting the imaging content. (**c**) First frame of the video, showing no prominent Ca^2+^ activity. (**d**) Frame at 53.15 s during recording, where the white box marks a region with clearly visible Ca^2+^ activity.

The procedures for viral injection, cranial window implantation, and in vivo two-photon Ca^2+^ imaging in mice were performed with reference to previously reported astrocytic imaging protocols [20,31,32]. The overall in vivo experimental procedure is illustrated in Figure 4.

All animal experiments were approved by the Institutional Animal Care and Use Committee (IACUC) of the university and were conducted in accordance with the relevant ethical guidelines (Animal Ethical Statement No. AMUWEC20230126). A total of seven male C57BL/6J mice (Jiangsu Jicui Yaokang Biotechnology Co., Ltd., Nanjing, China) aged 2–3 months were housed under standard conditions with controlled temperature and humidity, a 12 h light/dark cycle, and ad libitum access to food and water.

Under 1–1.5% isoflurane anesthesia (RWD Life Science, Shenzhen, China), stereotaxic injections were performed to deliver AAV5 encoding GfaABC1D-cytoGCaMP6f (Addgene, Watertown, MA, USA; Cat# 52925-AAV5) into the somatosensory cortex. For each injection site, 200–300 nL of viral solution (titer ≥ 7 × 10^12^ vg/mL) was delivered, the needle was left in place for 15 min after injection, and postoperative analgesia was administered. Approximately 4 weeks later, a chronic cranial window with a diameter of approximately 3 mm was implanted over the primary somatosensory cortex (S1; coordinates: AP + 0.5 mm, ML − 1.56 mm) at the previous injection site.

In vivo two-photon Ca^2+^ imaging was performed in cortical layers L1–L2/3 (typical imaging depth: ~80–180 μm) using a custom-built two-photon microscopy system equipped with a femtosecond pulsed laser (MaiTai, Spectra-Physics, Santa Clara, CA, USA), an electro-optic modulator (Model 350-80LA, Conoptics Inc., Danbury, CT, USA), a photomultiplier tube (H10770A-40, Hamamatsu Photonics, Hamamatsu, Japan), a resonant scanner (6SC2KA00-0Y, Cambridge Technology, Bedford, MA, USA), a galvanometric scanner (6SD11513, Cambridge Technology, Bedford, MA, USA), and a 40× water-immersion objective (Nikon, Tokyo, Japan). Continuous spontaneous calcium activity was recorded under 0.5–1% isoflurane anesthesia using an excitation wavelength of 920 nm and a frame rate of 40 Hz. The average laser power at the brain surface was adjusted to 15–30 mW based on imaging depth to avoid phototoxicity. During imaging, the head was fixed in a custom recording chamber, and the cortical surface was continuously perfused with warmed artificial cerebrospinal fluid.

## 3. Results

### 3.1. Performance on Simulated Datasets with Ground Truth

**Figure 5:**
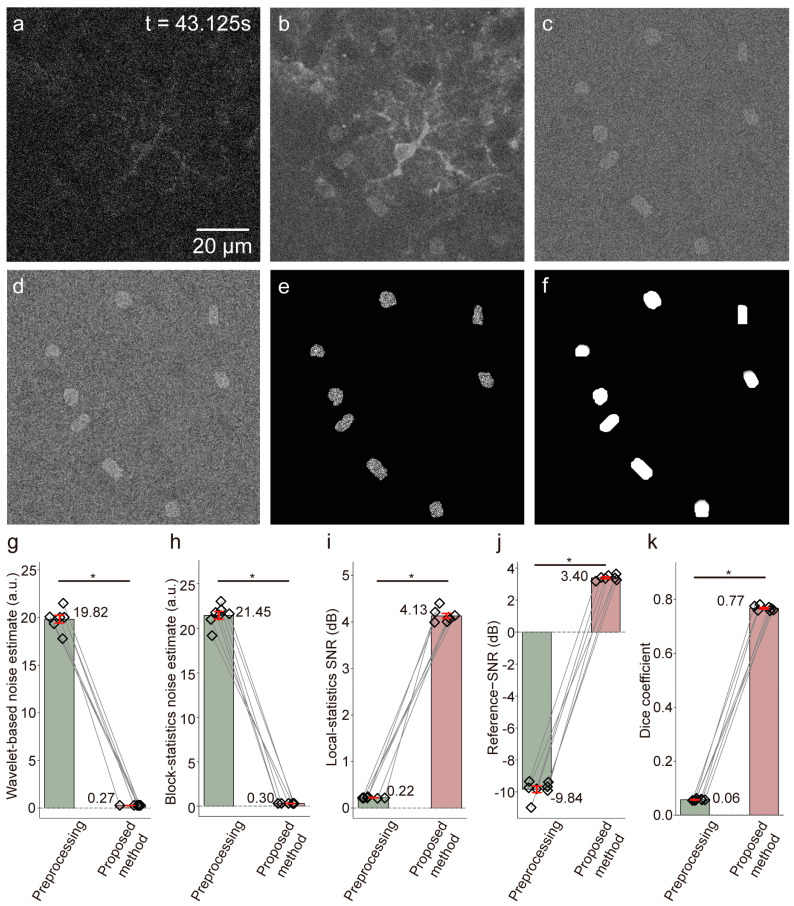
Extraction of dynamic calcium signals from simulated imaging using the proposed adaptive detection method. (**a**) Simulated two-photon calcium imaging of active astrocytic calcium signals (t = 43.125 s). (**b**) After frame accumulation. (**c**) After static background removal. (**d**) After Gaussian filtering. (**e**) Dynamic calcium signals extracted using the proposed adaptive dynamic signal detection method. (**f**) Program-generated dynamic calcium signals, serving as the ground truth for evaluating detection and segmentation performance. (**g**–**k**) Quantitative comparison of image quality metrics between preprocessing and the proposed method (*n* = 7 simulated videos). Bar plots show mean ± standard deviation for wavelet-based noise estimate (19.82 ± 1.02 vs. 0.27 ± 0.01 a.u.), block-statistics noise estimate (21.45 ± 1.09 vs. 0.30 ± 0.01 a.u.), local-statistics SNR (0.22 ± 0.01 vs. 4.13 ± 0.13 dB), reference SNR (−9.82 ± 0.50 vs. 3.40 ± 0.14 dB), and Dice coefficient (0.06 ± 0.01 vs. 0.77 ± 0.01). Statistical significance was assessed using the Wilcoxon matched-pairs signed rank test; * *p* < 0.05 indicates a significant difference.

We first evaluated the proposed framework on simulated two-photon datasets with event-level ground truth (*n* = 7 videos). As shown in Figure 5, preprocessing alone was insufficient to recover weak astrocytic calcium events from the simulated images. In contrast, the proposed framework substantially improved both image quality and segmentation performance, reducing the wavelet-based and block-statistics-based noise estimates to 0.27 ± 0.01 a.u. and 0.30 ± 0.01 a.u., respectively, while increasing the local-statistics-based SNR to 4.13 ± 0.13 dB, the reference SNR to 3.40 ± 0.14 dB, and the Dice coefficient to 0.77 ± 0.01. All improvements relative to preprocessing alone were statistically significant (Wilcoxon matched-pairs signed-rank test, two-sided, *p* < 0.05).

**Figure 6:**
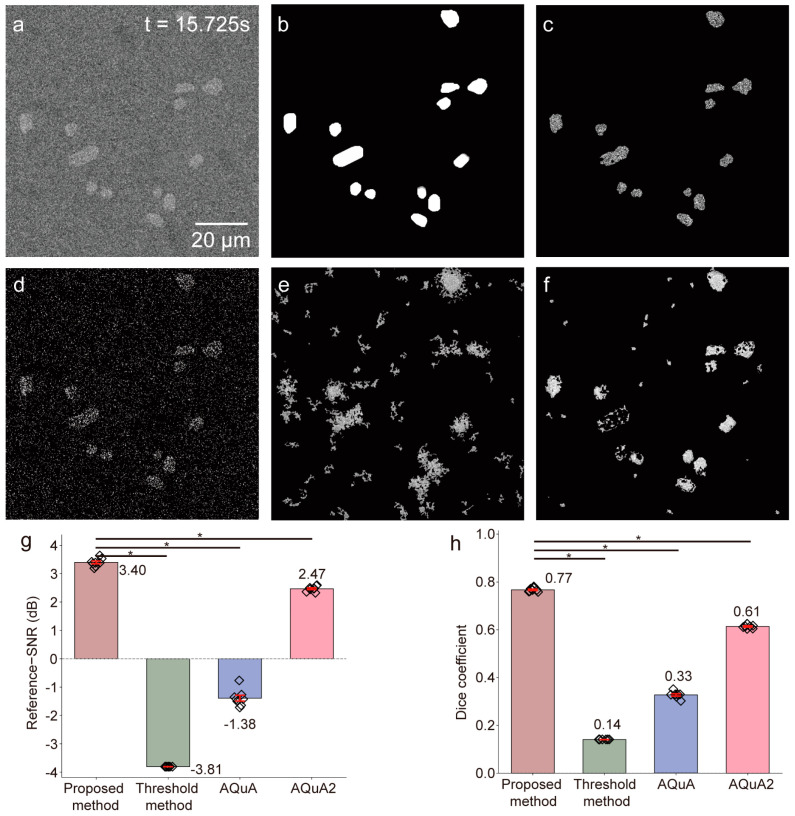
Comparison of calcium signal detection methods in simulated imaging. (**a**) Preprocessed image (t = 15.725 s). (**b**) Simulated active calcium signals (ground truth). (**c**) Proposed method. (**d**) Threshold method. (**e**) AQuA. (**f**) AQuA2. (**g**,**h**) Quantitative comparison of image quality metrics across methods (*n* = 7 simulated videos). Bar plots show mean ± standard deviation for reference SNR (proposed method: 3.40 ± 0.14, threshold method: −3.8 ± 0.02, AQuA: −1.38 ± 0.29, AQuA2: 2.47 ± 0.09 dB), and Dice coefficient (proposed method: 0.77 ± 0.01, threshold method: 0.14 ± 0.01, AQuA: 0.33 ± 0.01, AQuA2: 0.61 ± 0.01). Statistical significance was assessed using the Wilcoxon matched-pairs signed rank test, with Bonferroni correction for multiple comparisons; *p* < 0.05/3 ≈ 0.0167 indicates a statistically significant difference. Only pre-specified pairwise comparisons between the proposed method and the three baseline methods were performed; * *p* < 0.05 indicates a significant difference.

We next compared the proposed framework with a threshold-based method [33], AQuA [34], and AQuA2 [35] using the same preprocessed input images (Figure 6). The proposed framework achieved the best overall performance, with the highest reference SNR (3.40 ± 0.14 dB) and Dice coefficient (0.77 ± 0.01), compared with −3.80 ± 0.02 dB and 0.14 ± 0.01 for the threshold-based method, −1.38 ± 0.29 dB and 0.33 ± 0.01 for AQuA, and 2.47 ± 0.09 dB and 0.61 ± 0.01 for AQuA2. All pairwise comparisons between the proposed framework and the other methods were statistically significant (Wilcoxon matched-pairs signed-rank test, two-sided, *p* < 0.05).

Notably, AQuA and AQuA2 require substantially more tunable parameters than the proposed method (12/10 vs. 3) and exhibit greater sensitivity to parameter settings, which reduces their stability under high-noise conditions (see the Supplementary Materials for parameter details).

These results demonstrate that the proposed framework provides more accurate recovery of weak transient calcium events than preprocessing alone or the comparison methods in the simulated setting.

To further examine whether the Gaussian-mixture-based detection core depends critically on the Gaussian approximation in the presence of photon-limited noise, we additionally compared the proposed preprocessing pipeline with a photon-noise-aware variance-stabilizing strategy on the simulated Poisson–Gaussian datasets. Specifically, a generalized Anscombe transform (GAT) was inserted after frame accumulation and before background subtraction/normalization, while all downstream detection steps and user-defined parameters were kept unchanged. As shown in Supplementary Figure S5, the GAT-enhanced pipeline produced only marginal numerical changes relative to the proposed pipeline (Dice coefficient: 0.770 ± 0.011 vs. 0.767 ± 0.009; reference SNR: 3.43 ± 0.17 vs. 3.40 ± 0.15 dB; *n* = 7 simulated videos), and these differences were not statistically significant (two-sided Wilcoxon matched-pairs signed-rank test, both *p* = 0.1094). These results suggest that, under the present preprocessing conditions, the frame-wise intensity statistics are already sufficiently stabilized for effective downstream GMM-based signal–background decomposition, and that explicit photon-noise-aware variance stabilization provides only limited additional benefit.

### 3.2. Sensitivity Analysis of the Three User-Defined Parameters

**Figure 7:**
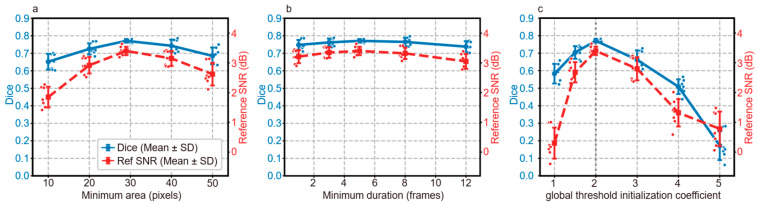
Sensitivity analysis of the three user-defined parameters in simulated datasets. (**a**) Effect of minimum event area (pixels) on Dice coefficient and reference SNR. (**b**) Effect of minimum event duration (frames) on Dice coefficient and reference SNR. (**c**) Effect of global threshold initialization coefficient on Dice coefficient and reference SNR. Solid lines show mean values, error bars represent standard deviation, and scattered points show individual results from seven simulated videos. Dashed vertical lines indicate the default parameter used in the main experiments.

To verify the robustness and usability of the proposed framework’s three user-defined parameters, we performed a systematic single-variable control sensitivity analysis. For each test, two parameters were fixed at their default values (minimum area = 29 pixels, minimum duration = 5 frames, global threshold initialization coefficient = 2.0), while the target parameter was varied across a physiologically and statistically relevant range. All tests were repeated independently across seven simulated calcium imaging videos, using Dice coefficient (segmentation accuracy) and reference SNR (signal retention quality) as core evaluation metrics (consistent with the full manuscript). Results are summarized in Figure 7.

For the minimum event area (Figure 7a), the framework achieved optimal performance at the default 29 pixels (Dice = 0.771, reference SNR = 3.40 dB). Within the physiological range of 10–40 pixels (covering astrocytic calcium microdomains to medium calcium waves), Dice coefficients remained above 0.7 (fluctuation < 6%). Thresholds < 20 pixels caused false positives from single-pixel noise, while thresholds > 40 pixels led to missed detection of small calcium microdomains, both degrading segmentation performance.

For the minimum event duration (Figure 7b), the framework exhibited the strongest robustness of the three parameters. Across 3–8 frames, Dice coefficients remained stable at 0.762–0.771 (maximum fluctuation < 1.2%), as the framework’s adaptive update mechanism balances the stability and sensitivity of Gaussian mixture modeling to dynamic calcium signals. Durations < 3 frames caused unstable segmentation from single-frame noise interference, while durations > 8 frames led to missed detection of fast transient calcium events due to insufficient temporal sensitivity.

For the global threshold initialization coefficient (Figure 7c), optimal performance was achieved at the default value of 2.0. Within the range of 1.5–3.0 (covering conventional threshold settings for calcium imaging), Dice coefficients remained between 0.704 and 0.771. Coefficients < 1.5 produced an overly permissive mask, introducing background noise that degraded Gaussian mixture modeling segmentation and increased false positives. Coefficients > 3.0 generated an overly strict mask that excluded weak calcium signals, causing severe missed detections and performance loss.

### 3.3. Image-Quality-Related Performance on In Vivo Two-Photon Recordings

**Figure 8:**
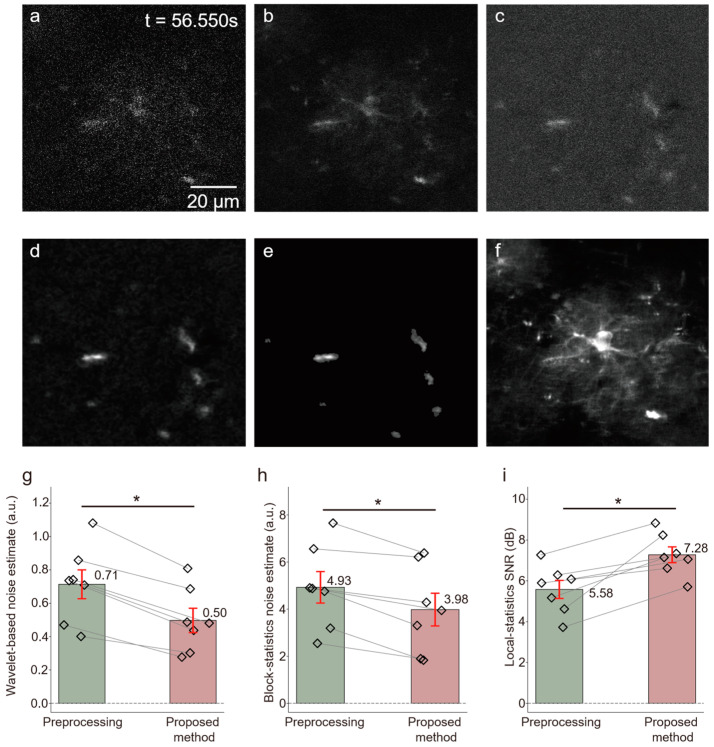
Results of dynamic signal extraction from in vivo two-photon calcium imaging of astrocytes. (**a**) Raw imaging frame (t = 2262 frame). (**b**) After frame accumulation. (**c**) After static background removal. (**d**) After Gaussian filtering. (**e**) Active calcium signals extracted using the proposed method. (**f**) Static background component from the raw imaging frame. (**g**–**i**) Quantitative comparison of image quality metrics between preprocessing and the proposed method (*n* = 7 mice). Bar plots show mean ± standard deviation for wavelet-based noise estimate (0.71 ± 0.21 vs. 0.50 ± 0.18 a.u.), block-statistics noise estimate (4.93 ± 1.64 vs. 3.98 ±1.70 a.u.), and local-statistics SNR (5.58 ± 1.08 vs. 7.28 ± 0.95 dB). Statistical significance was assessed using the Wilcoxon matched-pairs signed rank test; * *p* < 0.05 indicates a significant difference.

The proposed framework was further evaluated on in vivo two-photon calcium imaging recordings from seven mice (Figure 8). Compared with the simulated datasets, the in vivo recordings showed greater variability and stronger background coupling.

As shown in Figure 8, conventional preprocessing reduced part of the baseline fluctuation but did not fully resolve residual noise or blurred event boundaries. After application of the proposed framework, the wavelet-based noise estimate decreased from 0.71 ± 0.21 a.u. to 0.50 ± 0.18 a.u., the block-statistics-based noise estimate decreased from 4.93 ± 1.64 a.u. to 3.98 ± 1.70 a.u., and the local-statistics-based SNR increased from 5.58 ± 1.08 dB to 7.28 ± 0.95 dB (Figure 8g–i). All changes were statistically significant (Wilcoxon matched-pairs signed-rank test, two-sided, *p* < 0.05). Representative frames also showed clearer event boundaries and reduced background interference after adaptive detection. Because event-level ground truth was unavailable for the in vivo data, these results should be interpreted as improved image-quality-related performance rather than direct evidence of event-level detection accuracy. Similar validation logic has been used in other two-photon biological imaging studies under real tissue conditions. For example, Wang et al. applied two-photon fluorescence imaging in living cells and rat liver tissue slices, and interpreted fluorescence differences across control, injury, and treatment groups as biologically meaningful imaging readouts without relying on pixel-level segmentation labels [36]. Therefore, the present in vivo results should be understood as supportive real-data evidence under biologically relevant imaging conditions, rather than as direct event-level validation.

**Figure 9:**
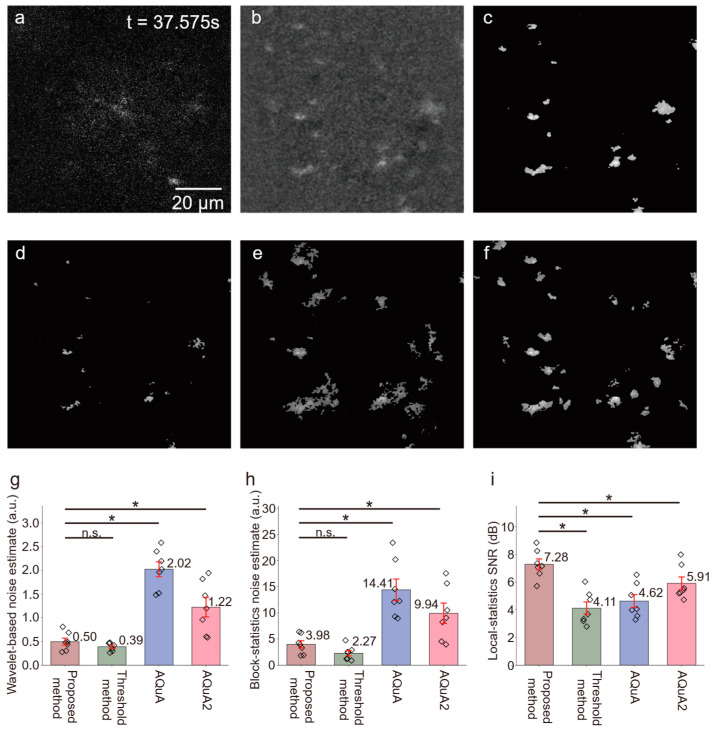
Comparison of calcium signal detection methods in in vivo two-photon imaging of astrocytes. (**a**) Raw image (t = 37.575 s). (**b**) Preprocessed image. (**c**) Active calcium signals detected by the proposed method. (**d**–**f**) Active calcium signals detected by the threshold method, AQuA, and AQuA2, respectively. (**g**–**i**) Quantitative comparison of image quality metrics through other methods (*n* = 7 mice). Bar plots show mean ± standard deviation for Wavelet-based noise estimate (proposed method: 0.50 ± 0.18, threshold method: 0.39 ± 0.03, AQuA: 2.02 ± 0.49, AQuA2: 1.22 ± 0.51 a.u.), Block-statistics noise estimate (proposed method: 3.98 ±1.70, threshold method: 2.27 ± 0.01, AQuA: 14.41 ± 4.67, AQuA2: 9.94 ± 4.78 a.u.) and Local-statistics SNR (proposed method: 7.28 ± 0.95, threshold method: 4.11 ± 1.14, AQuA: 4.62 ± 1.06, AQuA2: 5.91 ± 1.15 dB). Statistical significance was assessed using the Wilcoxon matched-pairs signed rank test, with Bonferroni correction for multiple comparisons; *p* < 0.05/3 ≈ 0.0167 indicates a statistically significant difference. Only pre-specified pairwise comparisons between the proposed method and the three baseline methods were performed; * *p* < 0.05 indicates a significant difference, and n.s. indicates no significant difference.

We further compared the proposed framework with the threshold-based methods AQuA and AQuA2 using the same preprocessed input images (Figure 9). The proposed framework achieved the highest local-statistics-based SNR among the evaluated methods, reaching 7.28 ± 0.95 dB, compared with 4.11 ± 1.14 dB for the threshold-based method, 4.62 ± 1.06 dB for AQuA, and 5.91 ± 1.15 dB for AQuA2. The proposed framework also produced markedly lower wavelet-based and block-statistics-based noise estimates than AQuA and AQuA2. Although the threshold-based method yielded lower estimated noise in some in vivo comparisons, it also produced substantially lower local SNR, suggesting that aggressive suppression may remove weak signal components together with noise.

Overall, these results indicate that the proposed framework achieved a more favorable balance between noise reduction and signal preservation under low-SNR in vivo imaging conditions.

To further quantify the contribution of each processing stage, we performed additional stage-wise ablation analyses across the raw input, frame accumulation, background subtraction and normalization, Gaussian smoothing, and the final adaptive detection stage. The corresponding results for the simulated and in vivo datasets are summarized in Supplementary Tables S1 and S2, respectively. In both datasets, the preprocessing steps mainly stabilized the input statistics and improved contrast, whereas the largest overall performance gain arose from the final adaptive detection stage.

## 4. Discussion

This study shows that the proposed adaptive threshold-based dynamic detection framework improves the extraction of weak astrocytic calcium signals in two-photon imaging. By combining segment-wise Gaussian mixture model (GMM) estimation, temporal masking, and adaptive threshold updating, the method improved both segmentation fidelity in simulated datasets and image-quality-related performance in vivo. In the simulated data, the framework substantially increased the Dice coefficient and reference SNR relative to preprocessing alone, whereas in vivo recordings showed reduced wavelet-based and block-statistics-based noise together with increased local SNR. These findings indicate that the proposed framework effectively suppresses heterogeneous noise while improving weak-signal recovery and segmentation fidelity for astrocytic calcium events. Therefore, the large Dice improvement observed in simulation should be interpreted as evidence of effective weak-signal recovery under a controlled benchmark with known ground truth, rather than as a claim that the simulated datasets fully capture all aspects of real astrocytic Ca^2+^ dynamics. An additional practical advantage is that the method requires only three user-defined parameters, which supports reproducible deployment across datasets with limited manual tuning.

Compared with conventional thresholding, the proposed framework provided more robust performance under heterogeneous noise and strong background coupling. Fixed-threshold approaches are prone to either incomplete signal recovery or excessive suppression, depending on the selected threshold, which can lead to blurred event boundaries or loss of weak signal components. In our in vivo comparisons, although the threshold-based method yielded lower estimated noise in some cases, it also showed substantially lower local SNR, suggesting that aggressive suppression may remove weak signal components together with noise. Relative to AQuA and AQuA2, the proposed framework achieved higher reference SNR and Dice coefficient in simulated data, and higher local SNR together with lower noise estimates in vivo. A likely reason is that AQuA-type pipelines depend more strongly on seed detection and onset-related spatiotemporal consistency, which may become unstable in low-SNR astrocytic recordings with strong coupling to static bright structures. In contrast, the proposed framework stabilizes intensity statistics before detection and explicitly enforces temporal persistence, making it less sensitive to noisy seed initialization under challenging imaging conditions. Compared with threshold-based methods, it preserves interpretability and low computational burden while replacing a static decision rule with data-adaptive local distribution modeling and temporal persistence constraints. Compared with AQuA and AQuA2, the present method focuses on an earlier but practically critical step—robust front-end extraction of weak candidates under heterogeneous noise and strong background coupling—rather than full event growth and propagation parsing. Compared with learning-based denoising approaches, it does not require training data, GPU-dependent optimization, or extensive hyperparameter search, and our supplementary comparison suggests that visually improved denoising does not necessarily translate into better downstream weak-signal detection. Thus, unlike threshold-based methods that prioritize simplicity, ROA-based pipelines such as STARDUST that prioritize regional feature extraction, or event-parsing frameworks such as AQuA, AQuA2, and astroCaST that prioritize downstream decomposition or clustering, the present method is intended primarily as an interpretable, low-parameter front-end solution for robust weak-signal recovery under heterogeneous noise and strong background coupling.

Among the components of the proposed framework, segment-wise GMM estimation is the principal element enabling the detection of weak astrocytic calcium signals, because it provides data-driven separation of signal-related peaks from heterogeneous background intensity distributions. However, GMM estimation alone is insufficient when signal prevalence and noise conditions vary over time. Therefore, adaptive threshold updating acts as the second key mechanism by dynamically regulating the decision boundary according to frame-level intensity statistics and inter-frame similarity. Temporal masking mainly functions as a stabilizing constraint that suppresses isolated fluctuations and improves temporal coherence, rather than serving as the primary detector by itself. The window-function transformation is used only for output mapping and display compatibility. Therefore, the key difference between the present work and previous methods is not the isolated use of any single module, but the explicit coupling of local distribution modeling, dynamic threshold control, and temporal persistence enforcement within a low-parameter framework for weak-signal detection. This coupled design provides a balance between noise suppression and transient-structure retention without relying on aggressive smoothing or training-intensive optimization. At the same time, the method remains conservative under extremely noisy conditions, and very small or short-lived events may be attenuated by the duration and area constraints as well as by the suppression-oriented design of the detection stage. This limitation reflects an inherent trade-off between false-positive control and weak-event sensitivity in low-SNR astrocytic imaging.

From a bioengineering perspective, the proposed framework offers several practical advantages. Its low parameter complexity reduces operator-dependent variability and facilitates reproducibility across experiments. Our systematic sensitivity analysis further demonstrates that, within their respective physiologically relevant ranges, changes to these three core parameters cause only limited fluctuations in detection performance. This high stability renders specialized manual fine-tuning unnecessary, effectively lowering the entry barrier for users without specialized image analysis experience. In addition, the method does not require paired training data, GPU-dependent model fitting, or extensive hyperparameter optimization, which distinguishes it from many deep-learning-based denoising approaches. These features make the framework attractive as an interpretable and computationally practical front-end module for large-scale experimental and preclinical calcium imaging workflows.

An additional point concerns the relationship between the Gaussian-mixture approximation and the photon-limited nature of two-photon imaging noise. In the present framework, the GMM is not intended as a physical model of the raw sensor noise, which is more appropriately described by Poisson or Poisson–Gaussian statistics. Rather, it is applied to the frame-wise intensity distribution after short-window accumulation, background subtraction/normalization, and Gaussian smoothing, that is, after the input statistics have been stabilized for downstream detection. Consistent with this interpretation, our supplementary comparison with a photon-noise-aware variance-stabilizing transform showed only marginal, statistically non-significant changes in Dice coefficient and reference SNR on the simulated Poisson–Gaussian datasets. This result supports the use of a Gaussian mixture as a practical empirical approximation for the preprocessed intensity distribution in the current framework, while also suggesting that photon-noise-aware preprocessing may provide a modest additional benefit in some settings.

Despite its robust performance under conventional astrocytic imaging conditions, the proposed framework has application limits. We characterized three extreme experimental scenarios where detection performance may decrease, with representative examples provided in Supplementary Figures S1–S3: (1) Severe non-rigid background drift, which compromises the spatiotemporal statistical consistency required for Gaussian mixture modeling; (2) Extremely low SNR, where signal intensity overlaps significantly with the noise floor, leading to missed detections; and (3) Spatiotemporal event overlap, such as large-scale calcium waves across multiple cells, which the current connected-component logic may merge into a single entity. It is important to note that these scenarios represent extreme or suboptimal experimental conditions (e.g., significant motion artifacts or improper laser power) rather than typical physiological astrocytic imaging.

This study also has several limitations. First, event-level ground truth was unavailable for the in vivo recordings; therefore, performance in real data was evaluated using image-quality-related metrics rather than direct event-detection accuracy. This type of supportive real-data validation is common in biologically complex two-photon imaging, where meaningful differences between experimental or pathological groups can provide external interpretive support even when pixel-level annotations are unavailable. For example, Wang et al. used two-photon imaging in living cells and liver tissue slices to distinguish between different biological states under real-tissue conditions, including control, injury, and treatment groups [36]. In this context, our in vivo validation should likewise be interpreted as a real-data assessment of robustness and signal interpretability, rather than as a fully annotated event-level benchmark. Second, validation was performed on simulated datasets and in vivo recordings from seven mice under the current imaging conditions, and broader evaluation across brain regions, imaging settings, indicators, and species will be necessary to establish generalizability. Third, the current CPU-based implementation may limit throughput for very large datasets. From a computational perspective, the main stages of the framework—including frame accumulation, background subtraction/normalization, Gaussian smoothing with a fixed kernel, temporal mask updating, connected-component filtering, and output mapping—scale approximately linearly with the number of processed pixels and frames under the present implementation settings. For segment-wise GMM estimation, the candidate model order is restricted to *K* = 1, 2, or 3, and the EM iteration number is capped at 100, ensuring that the per-update cost is effectively bounded by a small constant factor in practice. The memory requirement is mainly associated with frame-wise images, the background image, temporal count matrices, and binary masks, and does not involve trainable parameters, gradient storage, or intermediate feature maps as in deep-learning pipelines. Nevertheless, the current CPU-based implementation may still limit processing speed for very large datasets.

Therefore, future work will focus on expanding validation across more diverse datasets, improving computational efficiency through parallel or GPU-based implementations, and developing strategies to better preserve borderline-weak events in real-world low-SNR imaging. More broadly, computational approaches in neuroscience are expanding across multiple analytical scales, from image restoration in noisy biomedical imaging to dynamical modeling of pathological brain activity [37]. This trend further highlights the need for task-specific yet interpretable frameworks for astrocytic Ca^2+^ imaging analysis. We will investigate more adaptive temporal and spatial constraints, softer candidate-preservation mechanisms, and hybrid refinement strategies that combine the interpretability of statistical modeling with the adaptability of learning-based methods.

## 5. Conclusions

In conclusion, we present a low-parameter and interpretable detection framework for weak astrocytic Ca^2+^ signals in two-photon imaging. By combining segment-wise GMM estimation with temporal-coherence masking and adaptive threshold updates, our method improves segmentation fidelity in simulated data and enhances local signal prominence in in vivo recordings. Requiring only three user-defined parameters, this work provides a practical and computationally efficient front-end module to support reproducible analysis of low-SNR imaging data.

## 6. Patents

Tiangong University. Huiquan Wang, Jiameng Xu. Adaptive Threshold Segmentation Method and Apparatus for Astrocytic Dynamic Calcium Transients: CN121544658A [P]. 20 January 2026.

## Data Availability

The demonstration simulated data, analysis scripts, and code used in this study are publicly available at https://github.com/xujiameng/adaptive-gmm-calcium-detection. The raw in vivo two-photon imaging data presented in this study are available on reasonable request from the corresponding author.

## Supplementary Materials

The following supporting information can be downloaded at: https://www.mdpi.com/article/10.3390/bioengineering13050528/s1, Figure S1: Quantitative performance comparison of deep learning-based denoising methods for two-photon calcium imaging analysis; Figure S2: Severe non-rigid background drift; Figure S3: Extremely low-SNR weak-event attenuation; Figure S4: Spatiotemporal event overlap; Figure S5: Comparison between the proposed preprocessing pipeline and a photon-noise-aware variance-stabilizing strategy on simulated Poisson–Gaussian datasets; Table S1: Stage-wise ablation analysis of noise, SNR, and segmentation performance across simulated datasets (*n* = 7); Table S2: Stage-wise ablation analysis of noise and image-quality-related SNR across in vivo datasets (*n* = 7).

## Abbreviations

The following abbreviations are used in this manuscript:

GMM	Gaussian mixture model
SNR	Signal-to-noise ratio
ROI	Region of interest
MAD	Median absolute deviation
MSE	Mean squared error
IACUC	Institutional Animal Care and Use Committee
AAV	Adeno-associated virus
S1	Primary somatosensory cortex
