# Supplementary material for: A Low-Parameter Adaptive Framework Based on Gaussian Mixture Modeling for Detecting Weak Astrocytic Calcium Signals in Two-Photon Imaging
Source: Bioengineering (Basel). 2026 Apr 30;13(5):528. doi: 10.3390/bioengineering13050528 (PMC13203742; doi:10.3390/bioengineering13050528)
Supplement: Supplementary file 1 [file bioengineering-13-00528-s001.zip › bioengineering-4227755-supplementary.pdf]

# A Low-Parameter Adaptive Framework Based on Gaussian Mixture Modeling for Detecting Weak Astrocytic Calcium Signals in Two-Photon Imaging

## 1. Image preprocessing

### *1.1 Frame accumulation*

Images captured by two-photon microscopy are typically contaminated by additive noise that is spatially independent. To improve the signal-to-noise ratio (SNR) of weak signals, frame accumulation is employed. Compared with simply increasing exposure time—which may cause saturation and reduce dynamic range—multi-frame accumulation enhances SNR without changing sensor settings.

For the t-th frame, denote the image signal by $s_t$ , and assume that within a consecutive block of m frames the scene signal does not change significantly, denoted by $s$ Let $n_t$ be the random noise in the image, with noise variance $Var\left(n_t\right)=\sigma^2$ , and assume noise across frames is independent, i.e. $Cov\left(n_i,n_j\right)=0,i\neq j$ . The single-frame signal-to-noise ratio is expressed as $SNR_{\sin gle}=\frac{s}{\sigma}$. After accumulating m frames, the total signal is $\sum_{t=1}^{m}s_t=ms$ , and the total noise is $\sum_{t=1}^{m}n_t$ . The variance of the summed noise is $Var\left(\sum_{t=1}^{m}n_t\right)=\sum_{t=1}^{m}Var\left(n_t\right)=m\sigma^2$ , so the noise standard deviation is $\sqrt{m\sigma^2}=\sqrt{m}\sigma$. Therefore the SNR of the accumulated image is

$$SNR_{sum}=\frac{m\cdot s}{\sqrt{m}\sigma}=\sqrt{m}\frac{s}{\sigma}=\sqrt{m}SNR_{\sin gle}$$

Based on the average minimum calcium event duration of approximately 333 ms, and noting that an excessively long accumulation window may sum multiple distinct calcium events that overlap spatially—causing multiple events to be misidentified as a single event—, and given the original video sampling rate of 40 Hz, we set the frame accumulation window length to $0.333s\times40frame/s\approx13frame$, with a sliding step of 1 frame. To avoid pixel value overflow during frame accumulation, the original 8-bit grayscale video is converted to 16-bit format.

### *2.1 Background Subtraction and Normalization*

To decouple the static background from dynamic calcium signals, we adopt a “background subtraction and normalization” strategy. Let the original frame-accumulated video sequence be denoted as $F=\left\{f_t\left(x\right)\right\}_{t=1}^{T}$, $x\in\Omega$, where $\Omega\subset R^2$ represents the imaging field of view and $f_t\left(x\right)$ is the 16-bit gray value at pixel x in frame t. Subsequently, temporal averaging over all frames yields the background image $f_0\left(x\right)=\frac{1}{T}\sum_{t=1}^{T}f_t\left(x\right)$.

Because astrocytic scaffolds, blood vessels and similar structures remain almost invariant on the time scale, whereas calcium events are high-frequency and noise is symmetric, $f_0(x)$ retains predominantly static tissue signals. We then remove the static background frame-wise and normalize, obtaining a relative-change video that contains only dynamic variations plus noise:

$$\Delta_t(x) = \frac{f_t(x) - f_0(x)}{f_0(x) + \varepsilon}$$

, where $\varepsilon = 1$ prevents division by zero.

*1.3 Gaussian filter*

At the final step of preprocessing, a Gaussian filter is applied to the original 16-bit imaging sequence for spatio-temporal smoothing, so as to suppress high-frequency noise and reduce baseline fluctuations. Treating frame t, $F_t(x, y)$ , as a continuous function, we convolve it with an isotropic Gaussian kernel whose function is: $G_\sigma(u,v) = \frac{1}{2\pi\sigma^2}\exp\left(-\frac{u^2+v^2}{2\sigma^2}\right)$. where u and v is the pixel-coordinate offset, $\sigma$ controls the smoothing strength, and $2\pi\sigma^2$ is the normalization constant ensuring $\iint G_\sigma(u,v)\,dudv = 1$ . To avoid truncation error, the convolution window radius $R$ is set by $R = k\sigma, k = 4$, covering $(2R+1)^2$ pixels in total. The discrete convolution formula is:

$$\hat{F}_t(i,j) = \sum_{u=-R}^{R}\sum_{v=-R}^{R} \omega_{u,v} F_t(i+u, j+v)$$

, where $\omega_{u,v} = \frac{G_\sigma(u,v)}{\sum_{u'=-R}^{R}\sum_{v'=-R}^{R} G_\sigma(u',v')}$ is adopted at boundaries to prevent edge artifacts.

In this paper $\sigma$ is set to 1.0.

## 2. Evaluation Metrics

*2.1 Noise Estimation*

*2.1.1 Local-Statistics-Based SNR*

Suppose the entire video consists of T grayscale frames and denote frame t as $I_t(x, y)$ . Divide this frame into non-overlapping macro-blocks $\Omega_{t,i}$ of size $B \times B$ , the local variance $\sigma^2_{\Omega_{t,i}} = \frac{1}{B^2}\sum_{(x,y)\in\Omega_{t,i}}\left(I_t(x,y) - \overline{I}_{\Omega_{t,i}}\right)^2$ ,where $\overline{I}_{\Omega_{t,i}}$ is the mean intensity of block $\Omega_{t,i}$ . Astrocytic calcium events are sparse and energetically concentrated, so the corresponding blocks exhibit variances significantly higher than those containing only noise. We retain the flattest 10 % of the variance distribution, and take the median of

their variances as the noise-level estimate for frame t: $\hat{\sigma}_{BEV}^{(t)} = \sqrt{median\left\{\sigma_{\Omega_{t,i}}^{2}\right\}_{i=1}^{N_{flat}^{(t)}}}$ ,

where $N_{flat}^{(t)}$ is the number of blocks identified as "flat." In the experiments we set B = 8 pixels, trading off estimation accuracy against computational efficiency.

After traversing all frames, the final noise estimate is obtained by averaging:

$$\sigma_{BEV} = \frac{1}{T}\sum_{t=1}^{T}\hat{\sigma}_{BEV}^{(t)}$$

*2.1.2 Wavelet Transform Method*

Wavelet decomposition splits an image into sub-bands of different scales and orientations; among them, the highest-frequency diagonal sub-band contains almost no structural information and is the main locus of noise energy. Exploiting this property, the noise standard deviation can be robustly estimated without any reference.

For frame t , denoted $I_t(x,y)$ , perform one-level discrete wavelet decomposition using the orthogonal Daubechies-1 wavelet (db1): $I_t(x,y)\xrightarrow{DWT}\{LL_1, LH_1, HL_1, HH_1\}$ , where DWT(Discrete Wavelet Transform)； $LL_1$ : low–low band, preserving the main contour; $LH_1$ : low–high band, capturing horizontal edges/texture; $HL_1$为: high–low band, capturing vertical edges/texture; $HH_1$ : high–high band, almost signal-free but rich in noise—the key region for noise estimation. Denote $\omega$ as all coefficients of $HH_1$ then the noise-level estimate for frame t is $\hat{\sigma}_{WRME}^{(t)} = \frac{median(|\omega|)}{0.6745}$, where the divisor 0.6745 ensures the estimator is unbiased and asymptotically consistent for zero-mean Gaussian noise.

After traversing all frames, the final wavelet-based noise estimate is obtained by averaging:

$$\sigma_{WRME} = \frac{1}{T}\sum_{t=1}^{T}\hat{\sigma}_{WRME}^{(t)}$$

## 2.2 Signal to Noise Ratio

*2.2.1. Local-Statistics-Based SNR*

For frame-t image $I_t(x,y)$, an $11\times11$ box filter is adopted. This odd size is about twice the minimum calcium-signal length (~5×5 pixels), so the window can cover a single event while introducing little background interference. The uniform filter is $B(x,y) = \frac{1}{121}\sum_{u,v\in(x,y)} I(u,v)$ , where $(x,y)$ is the 11×11 neighborhood. The per-pixel residual is then $N(x,y) = I(x,y) - B(x,y)$ , and the local-statistics-based signal-to-noise ratio is:

$$SNR_{local} = 10\log_{10}\left(\frac{\sum_{x,y} I(x,y)^2}{\sum_{x,y} B(x,y)^2}\right)$$

*2.2.2. Reference SNR*

For synthetic images, a "ground truth" is available; thus, the signal-to-noise ratio is defined via the mean-square-error power ratio.

Let two grayscale frames of the same size be given for frame t: the ground-truth frame $I_{true}(x,y)$ and the estimated frame $I_{est}(x,y)$. The mean-square error is $MSE = \frac{1}{HW}\sum_{x,y}\left[I_{true}(x,y) - I_{est}(x,y)\right]^2$, where H and W are the height and width of the frame, respectively. The power of the ground-truth frame is $P_{signal} = \frac{1}{HW}\sum_{x,y} I_{true}(x,y)^2$. Then, for synthetic images, the SNR against the ground-truth frame is defined as:

$$SNR_{ref} = 10\log_{10}\left(\frac{P_{signal}}{MSE}\right)$$

*2.2.3 Dice coefficient*

To quantitatively evaluate the spatiotemporal consistency between the pre-processed simulated signals and the real active calcium events, the Dice Similarity Coefficient (DSC) is employed as the evaluation metric. DSC is a widely used overlap measure in the field of image segmentation, defined as twice the size of the intersection of two binary sets divided by the sum of their sizes. The calculation process for the Dice coefficient of frame t is as follows:

$$Dice_t = \frac{2|A_t \cap B_t|}{|A_t| + |B_t|}$$

Where $A_t = \{(x,y) \mid G_t(x,y) > 0\}$ denotes the spatial mask of active calcium signals in frame t of the simulated video; $B_t = \{(x,y) \mid I_t(x,y) > 0\}$ denotes the spatial mask in frame t of the pre-processed video.

The Dice coefficient ranges from 0 to 1, with a higher value indicating greater similarity between the sets and thus more accurate segmentation. A Dice coefficient of 0 indicates no overlap, while a value of 1 indicates identical sets.

## 3. Simulation of Imaging Data

### *3.1 Gray-Level Normalization and Background Superposition*

For each frame of the computer-generated moving-spot images, the dynamic signal $F_{Ca}(x,y,t)$ was normalized to the range [0, 1] and linearly superimposed with the normalized cellular skeleton image $F_{Sk}(x,y)$ using a weighting coefficient:

$$F_{comb}(x,y,t) = \omega_{Ca} \cdot F_{Ca}(x,y,t) + \omega_{Sk} \cdot F_{Sk}(x,y,t)$$

where $\omega_{Ca} = 0.2$, $\omega_{Sk} = 0.8$. It preserves the occlusion effect of the skeleton while preventing an excessively strong background from overwhelming weak signals.

*3.2 Noise Model*

Three major types of camera noise were introduced into the superimposed image sequence:：

a. Dark current noise (Poisson-distributed)

$$N_{dark} \sim \text{poisson}(\lambda_{dark})$$

, where $\lambda_{dark} = 0.9e^{-} / pixel / frame$

b. Shot noise (Poisson-distributed)

$$N_{shot} \sim \text{poisson}(F_{comb})$$

c. Read noise (Gaussian-distributed)

$$N_{read} \sim N(0, \sigma_{read}^{2}), \text{ where } \sigma_{read} = 0.4e^{-}$$

*3.3 Noise Synthesis*

The above noise sources and the signal were combined according to the camera gain ( $g = 1.5DN / e^{-}$ ):

$$F_{noisy} = g \cdot [F_{comb} + N_{shot} + N_{dark} + N_{read}]$$

This process embedded dynamic calcium events into realistic morphological backgrounds and added noise with statistical properties consistent with actual imaging, thereby generating a controlled and reproducible simulated dataset for subsequent algorithm evaluation.

**4. Parameter settings of comparative methods**

All parameters of method were manually tuned.

(1) This study: Minimum area threshold = 29 pixels (in our two-photon calcium imaging, 1 pixel = 0.187 μm); minimum signal duration = 5 frames (1 frame = 0.025 s, 40 Hz);

(2) Conventional Threshold Method: Threshold set to $mean + k \cdot std$， where $k = 2$， It was chosen to maximize the Dice coefficient.

These parameter values were selected based on imaging resolution, expert experience, and common practice in calcium imaging analysis. The minimum duration and area thresholds reflect the minimal temporal and spatial scales considered physiologically meaningful for astrocytic $Ca^{2+}$ activity under our two-photon imaging conditions, while the global threshold initialization value follows commonly used intensity-based detection settings and is subsequently refined by adaptive updating.

All comparative methods were implemented using their official public releases, and the source code was not modified in this study. AQuA was implemented in MATLAB 2018 using the official MATLAB source code from the Yu Lab repository (https://github.com/yu-lab-vt/AQuA). AQuA2 was implemented in MATLAB 2025 using the official MATLAB source code from the Yu Lab repository (https://github.com/yu-lab-vt/AQuA2). Parameter optimization and dataset adaptation for both baselines were performed according to the official user guides and parameter documentation provided by the developers. Specifically, only dataset-dependent threshold-scaling-related settings were adjusted between simulated and in vivo two-photon data when necessary, whereas the remaining parameters were selected within methodologically and physiologically appropriate ranges and then kept fixed for quantitative comparison. The final settings used in this study are listed below:

(3) AQuA Parameters: Smoothing (sigma)=1, Minimum size (pixels)=29, Temporal cut threshold=2, Growing z threshold=1, Rising time uncertainty=2, Slowest delay in propagation=2, Propagation smoothness=1, Z score threshold=2, Maximum distance=0, Minimum correlation=0, Maximum time difference=5

(4) AQuA2 Parameters: Median fiiter radius (For salt and pepper noise)=2, Gaussian filter radius=1, Intensity threshold scaling factor=10/17 (simulation/two-photon data), Minimum duration=5, Minimum size (pixels) =29, Minimum seed size / active region=0.01, Zscore of seed significance=3.5, Maximum dissimilarity allowed in merging=0.6, Minimum source size / super event=0.01, Sensitivity to detect source (Level 1 to 10)=8.

## 5. Quantitative Comparison with Deep Learning-Based Denoising Methods

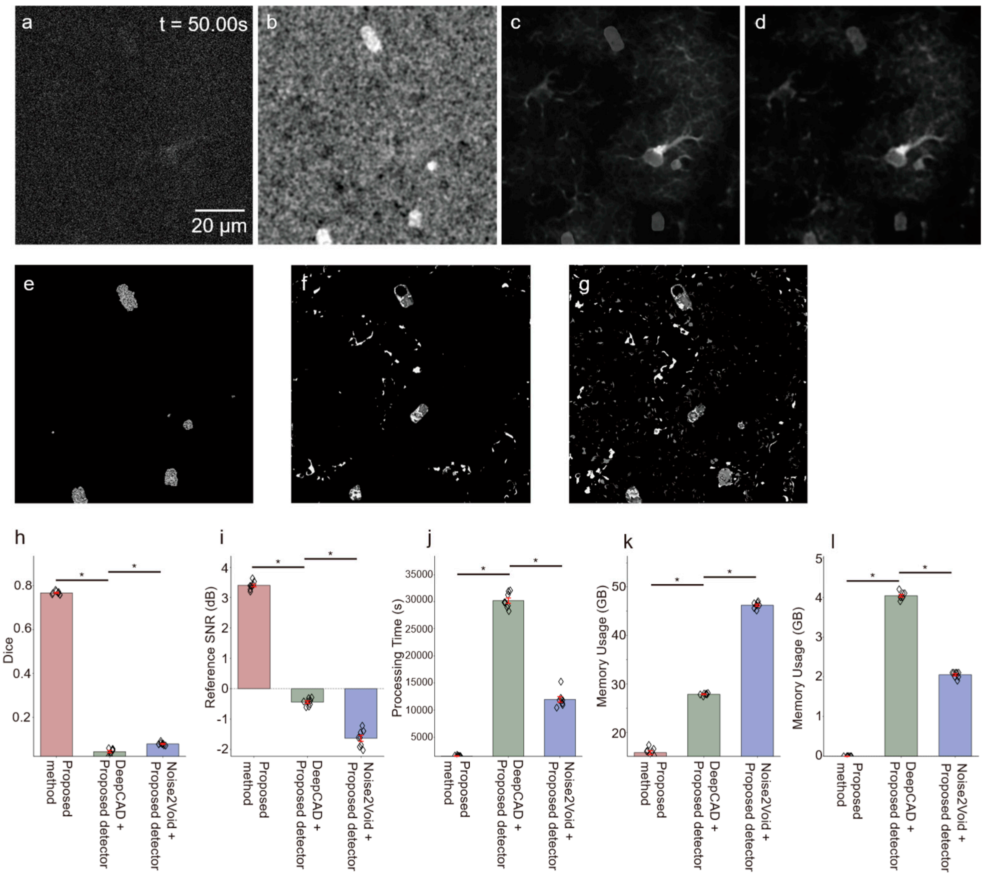


**Figure S1.** Quantitative performance comparison of deep learning-based denoising methods for two-photon calcium imaging analysis. (a) Raw simulated two-photon calcium imaging frame. (b) Preprocessed frame from the proposed method. (c) Denoised frame from DeepCAD. (d) Denoised frame from Noise2Void. (e) Calcium signal detection result based on the proposed preprocessing pipeline. (f) Calcium signal detection result based on DeepCAD-denoised frames. (g) Calcium signal detection result based on Noise2Void-denoised frames. (h-l) Quantitative comparison of detection performance and computational cost across methods: (h) Dice coefficient, (i) Reference SNR (dB), (j) Total processing time (s), (k) Memory usage (GB), (l) GPU memory usage (GB). Data are presented as mean ± standard deviation (n=7 simulated videos). Statistical significance was assessed using the Wilcoxon matched pairs signed rank test, with Bonferroni correction for multiple comparisons; $p < 0.05/2 = 0.025$ indicates statistically significant difference. Only pre-specified pairwise comparisons between the proposed method and the two baseline methods were performed. All experiments were performed on a workstation with the following configuration: Hardware: Intel(R) Xeon(R) CPU E5-2666 v3 @ 2.90 GHz, 128 GB RAM, NVIDIA GeForce RTX 3060 GPU (12 GB VRAM); Software: Python 3.11, PyTorch 2.0.1, CUDA 11.8.

Under fixed parameter settings, the computational cost of the proposed framework grows approximately linearly with the number of video pixels and frames. Specifically, frame accumulation, background subtraction/normalization, temporal mask updating, connected-component filtering, and the final window-function mapping are all frame-wise operations with complexity proportional to the number of processed pixels. Gaussian smoothing also remains linear in practice because the kernel size is fixed in

this study ( σ = 1.0). For segment-wise GMM estimation, the model order is restricted to K = 1, 2, or 3 and the EM iteration number is capped at 100; therefore, its per-update cost is also effectively linear in the number of pixels, with a bounded constant factor. Accordingly, the overall time complexity can be approximated as linear in the input data size under the present implementation settings.

As shown in Figure S2, the proposed framework achieved significantly higher segmentation accuracy and signal retention than pipelines using deep learning-based denoising preprocessing. The proposed method yielded a mean Dice coefficient of 0.77 ± 0.01 and reference SNR of 3.40 ± 0.14 dB, compared with 0.04 ± 0.02 and -0.45 ± 0.12 dB for DeepCAD, and 0.08 ± 0.01 and -1.63 ± 0.30 dB for Noise2Void. Meanwhile, the proposed method had substantially lower computational consumption: processing speed was ~20× faster than DeepCAD and ~8× faster than Noise2Void, with no GPU memory required. The poor detection performance of deep learning pipelines was attributed to denoising-induced artificial artifacts, which interfered with accurate identification of real calcium signals.

Parameter Settings for Deep Learning-Based Denoising Methods as following: (1) The DeepCAD-RT self-supervised denoising model was built on a 3D U-Net architecture with 16 feature map channels. The model was trained for 30 epochs using the Adam optimizer ($\beta_1$=0.5, $\beta_2$=0.999) with an initial learning rate of 1e-5. Training patches were set to 110×110×110 (T×H×W) with an overlap factor of 0.25, and the total training dataset included 2999 3D patches. (2) The Noise2Void self-supervised denoising model was built on a 2D U-Net architecture with 32 base convolution channels. The model was trained for 30 epochs using the Adam optimizer with a learning rate of 1e-5, and the MSE loss function was used for optimization. Training patches were set to 64×64 with a batch size of 16, and at least 50 patch sampling iterations were performed per epoch.

## 6. Representative cases of the extreme conditions.

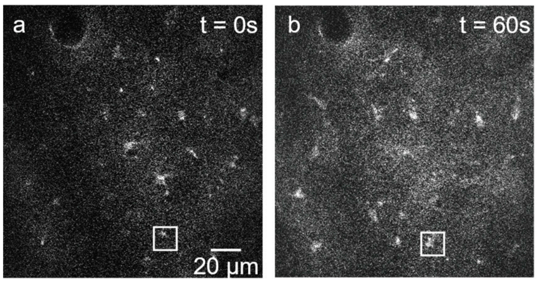


**Figure S2.** Severe non-rigid background drift. (a) Two-photon calcium imaging acquired at the beginning of the recording. (b) Two-photon calcium imaging acquired after the background drift. White boxes highlight the cellular shift within the field of view.

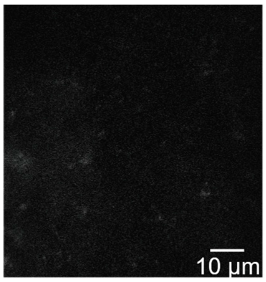


**Figure S3.** Extremely low-SNR weak-event attenuation

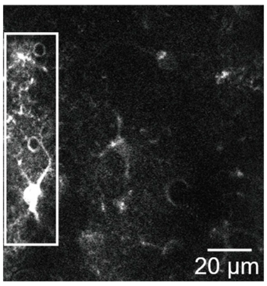


**Figure S4.** Spatiotemporal event overlap. White boxes indicate the simultaneous occurrence of multiple calcium events within the same spatiotemporal domain

## 7. Comparison with a Photon-Noise-Aware Variance-Stabilizing Transform

To evaluate whether a photon-noise-specific preprocessing strategy would materially improve downstream detection, we compared the proposed preprocessing pipeline with a generalized Anscombe transform (GAT)-enhanced branch on the 7 simulated datasets generated with Poisson–Gaussian noise. In the GAT-enhanced branch, GAT was inserted after frame accumulation and before background subtraction/normalization, while Gaussian smoothing, temporal masking, segment-wise GMM estimation, adaptive threshold updating, and all user-defined parameters were kept unchanged. Detection performance was evaluated using the Dice coefficient and reference SNR, consistent with the main manuscript for simulated data. Statistical significance was assessed using the two-sided Wilcoxon matched-pairs signed-rank test.

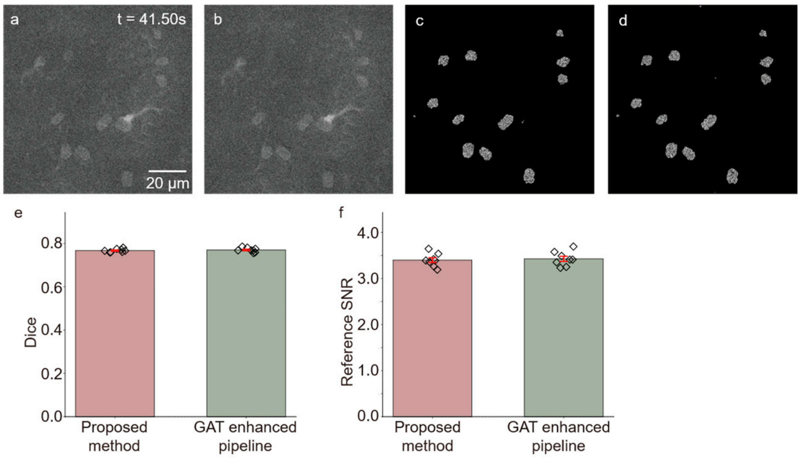

**Figure S5.** Comparison between the proposed preprocessing pipeline and a photon-noise-aware variance-stabilizing strategy on simulated Poisson–Gaussian datasets. (a) Representative simulated two-photon calcium imaging frame after the proposed preprocessing pipeline. (b) Representative simulated frame after insertion of a GAT after frame accumulation and before background subtraction/normalization. (c) Calcium signal detection result obtained with the proposed pipeline. (d) Calcium signal detection result obtained with the GAT-enhanced pipeline. (e–f) Paired quantitative comparison across 7 simulated videos for (e) Dice coefficient and (f) reference SNR (dB). Data points represent individual videos; lines connect paired measurements from the same simulated video. The GAT-enhanced pipeline showed slightly higher mean Dice coefficient and reference SNR than the proposed pipeline, but the differences were not statistically significant (two-sided Wilcoxon matched-pairs signed-rank test: Dice, $p=0.1094$; reference SNR, $p=0.1094$). These results indicate that, under the present preprocessing and imaging conditions, explicit photon-noise-aware variance stabilization provides only a limited additional benefit to the downstream detection performance.

## 8. Stage-Wise Ablation Analysis of the Preprocessing Pipeline and Final Adaptive Detection

**Table S1.** Stage-wise ablation analysis of noise, SNR, and segmentation performance across simulated datasets (n = 7).

| Simulated datasets (n=7) | Raw data | Frame accumulation | Background subtraction and normalization | Gaussian smoothing (preprocessing) | Proposed method |
|---|---|---|---|---|---|
| Wavelet-based noise | 27.5 ± 2.0 | 8.8 ± 0.7 | 23.5 ± 2.2 | 19.82 ± 1.10 | 0.270 ± 0.000 |
| Block-statistics noise | 25.5 ± 1.6 | 7.4 ± 0.6 | 23.8 ± 1.8 | 21.45 ± 1.18 | 0.300 ± 0.000 |
| Local-statistics SNR (dB) | 0.12 ± 0.02 | 0.90 ± 0.12 | 0.18 ± 0.04 | 0.22 ± 0.01 | 4.13 ± 0.14 |
| Reference-SNR (dB) | -13.5 ± 0.9 | -11.8 ± 0.8 | -10.6 ± 0.7 | -9.84 ± 0.55 | 3.40 ± 0.15 |
| Dice coefficient | 0.015 ± 0.006 | 0.025 ± 0.008 | 0.040 ± 0.010 | 0.057 ± 0.003 | 0.767 ± 0.009 |

For the simulated datasets, frame accumulation provided the first substantial reduction in both wavelet-based and block-statistics-based noise, together with modest improvements in local-statistics SNR, reference SNR, and Dice coefficient. Background subtraction and normalization reduced static background coupling, but also caused a rebound in noise estimates, consistent with fluctuation amplification after relative-change normalization. Gaussian smoothing provided additional stabilization of the input statistics, but preprocessing alone remained insufficient for accurate weak-signal recovery. The largest overall improvement occurred only after the final adaptive detection stage, where all metrics improved sharply, indicating that preprocessing mainly serves a supporting role, whereas the dominant performance gain arises from the coupled detection core.

**Table S2.** Stage-wise ablation analysis of noise and image-quality-related SNR across in vivo datasets (n = 7).

| In vivo datasets (n=7) | Raw data | Frame accumulation | Background subtraction and normalization | Step3: Gaussian smoothing (preprocessing) | Proposed method |
|---|---|---|---|---|---|
| Wavelet-based noise | 1.10 ± 0.35 | 0.85 ± 0.28 | 0.95 ± 0.30 | 0.714 ± 0.229 | 0.497 ± 0.193 |

| | | | | | |
|---|---|---|---|---|---|
| Block-statistics noise | 6.8 ± 2.1 | 5.6 ± 1.8 | 5.9 ± 1.9 | 4.93 ± 1.77 | 3.98 ± 1.84 |
| Local-statistics SNR (dB) | 1.4 ± 0.6 | 2.6 ± 0.8 | 2.0 ± 0.7 | 5.58 ± 1.17 | 7.28 ± 1.03 |

For the in vivo datasets, a similar stage-wise pattern was observed. Frame accumulation reduced noise and increased local-statistics SNR, whereas background subtraction and normalization produced a mild metric rebound, likely due to enhanced sensitivity to local baseline fluctuation. Gaussian smoothing further stabilized the signal statistics, and the final proposed method achieved the lowest noise estimates and the highest local-statistics SNR. Because event-level ground truth was unavailable for the in vivo data, these results should be interpreted as progressive image-quality-related improvement rather than direct evidence of event-level detection accuracy.
